# Metformin as a new anti-cancer drug in adrenocortical carcinoma

**DOI:** 10.18632/oncotarget.10421

**Published:** 2016-07-06

**Authors:** Giada Poli, Giulia Cantini, Roberta Armignacco, Rossella Fucci, Raffaella Santi, Letizia Canu, Gabriella Nesi, Massimo Mannelli, Michaela Luconi

**Affiliations:** ^1^ Endocrinology Unit, Department of Experimental and Clinical Biomedical Sciences “Mario Serio”, University of Florence, Florence Italy; ^2^ Division of Pathological Anatomy, Department of Surgery and Translational Medicine, University of Florence, Florence Italy

**Keywords:** metformin, H295R, IGF-1R, apoptosis, tumor proliferation

## Abstract

Adrenocortical carcinoma (ACC) is a rare heterogeneous malignancy with poor prognosis. Since radical surgery is the only available treatment, more specific and effective drugs are urgently required. The anti-diabetic drug metformin has been associated with a decreased cancer prevalence and mortality in several solid tumors, prompting its possible use for ACC treatment.

This paper evaluates the *in vitro* and *in vivo* anti-cancer effects of metformin using the ACC cell model H295R.

Metformin treatment significantly reduces cell viability and proliferation in a dose- and time-dependent manner and associates with a significant inhibition of ERK1/2 and mTOR phosphorylation/activation, as well as with stimulation of AMPK activity. Metformin also triggers the apoptotic pathway, shown by the decreased expression of Bcl-2 and HSP27, HSP60 and HSP70, and enhanced membrane exposure of annexin V, resulting in activation of caspase-3 apoptotic effector. Metformin interferes with the proliferative autocrine loop of IGF2/IGF-1R, which supports adrenal cancer growth. Finally, in the ACC xenograft mouse model, obtained by subcutaneous injection of H295R cells, metformin intraperitoneal administration inhibits tumor growth, confirmed by the significant reduction of Ki67%.

Our data suggest that metformin inhibits H295R cell growth both *in vitro* and *in vivo*. Further preclinical studies are necessary to validate the potential anti-cancer effect of metformin in patients affected by ACC.

## INTRODUCTION

Despite its rarity (1:2 million prevalence), adrenocortical cancer (ACC) deserves more consideration on account of its aggressive behavior and poor prognosis when metastatic at diagnosis. To date, the best curative option is radical surgery. In case of advanced ACC, mitotane is the only available medical treatment [1, 2]. Nevertheless, mitotane has proved to be of limited efficacy and poor tolerability, thus often reducing the patient's compliance. The drug's therapeutic range is reached in less than 50% of treated patients [1]; moreover, specific polymorphisms of the CYP2B6 gene have been associated with reduced circulating levels of mitotane, further decreasing its efficacy [3]. The mechanism by which mitotane acts on cancer cells, as well as on the normal adrenal, is still far from being fully elucidated. In such a scenario, it is mandatory to combine mitotane with other drugs, in order to improve the therapeutic efficacy and reduce treatment toxicity. Of the possible drugs that could be associated with mitotane, those safely and widely administered for treating other diseases, and eventually found to also exert anti-neoplastic effects, should offer the best therapeutic option. Metformin (1,1-dimethylbiguanide) is a biguanide cationic compound commonly used as an insulin-sensitizer and glucose-lowering drug in the treatment of type 2 diabetes (T2D). Epidemiological studies and meta-analyses on large cohorts of diabetic patients have demonstrated a significant association between metformin and a reduced incidence of various types of solid tumors [4–7], supporting the potential use of metformin as an anti-cancer drug [8]. Several clinical trials are currently ongoing to specifically test metformin efficacy in cancer prevention and therapy [9, https://clinicaltrials.gov]. A considerable number of studies have demonstrated the anti-cancer activity of this drug both in *in vitro* and *in vivo* tumor models, highlighting a direct anti-proliferative and pro-apoptotic effect on cancer cells and an indirect action on metabolic regulation [8, 9].

The present paper investigates the *in vitro* and *in vivo* effects of metformin on the H295R adrenocortical cancer cell line.

## RESULTS

### Metformin inhibits cell viability and proliferation in H295R cells

**Figure 1:**
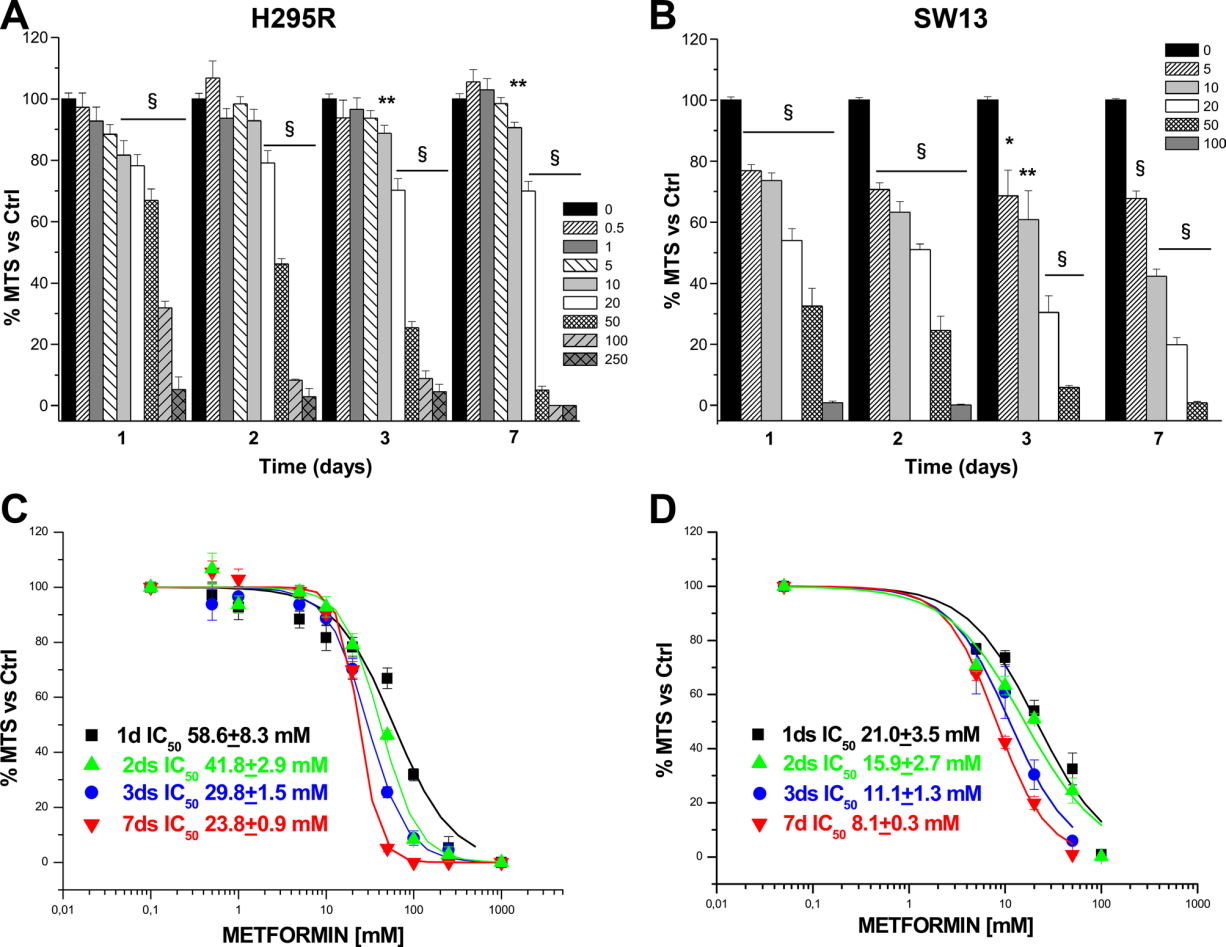
Metformin inhibits H295R and SW13 cell viability Cell viability was assessed by using MTS assay in H295R (**A**) and SW13 (**B**) cells grown in the presence or absence of increasing doses of metformin (mM) at the indicated time points. Data are expressed as mean ± SE of absorbance percentage vs. non-stimulated controls in *n* = 5 independent experiments. Statistical analysis was performed with ANOVA followed by Dunnett's post hoc test: *P < 0.05, **P < 0.01, ^§^*P* < 0.0001 vs respective controls. Metformin IC50s for cell viability were calculated on the dose-response curves obtained at the indicated time points in H295R (**C**) and SW13 (**D**) cell lines.

To investigate the effects of metformin on ACC, we first evaluated whether metformin interfered with viability in two available ACC cell lines, H295R and SW13. *In vitro* administration of increasing doses of metformin resulted in a dose- and time-dependent decrease of cell viability, which was statistically significant starting from 24 hours, as assessed by MTS assay in both the H295R (Figure 1A) and SW13 (Figure 1B) cell lines. Analysis of MTS dose-response curves allowed calculation of metformin inhibitory half doses (IC_50_) for viability. Comparison of the IC_50_s results revealed that the drug had a stronger effect on SW13 than H295R cells (Figure 1C, 1D).

**Figure 2:**
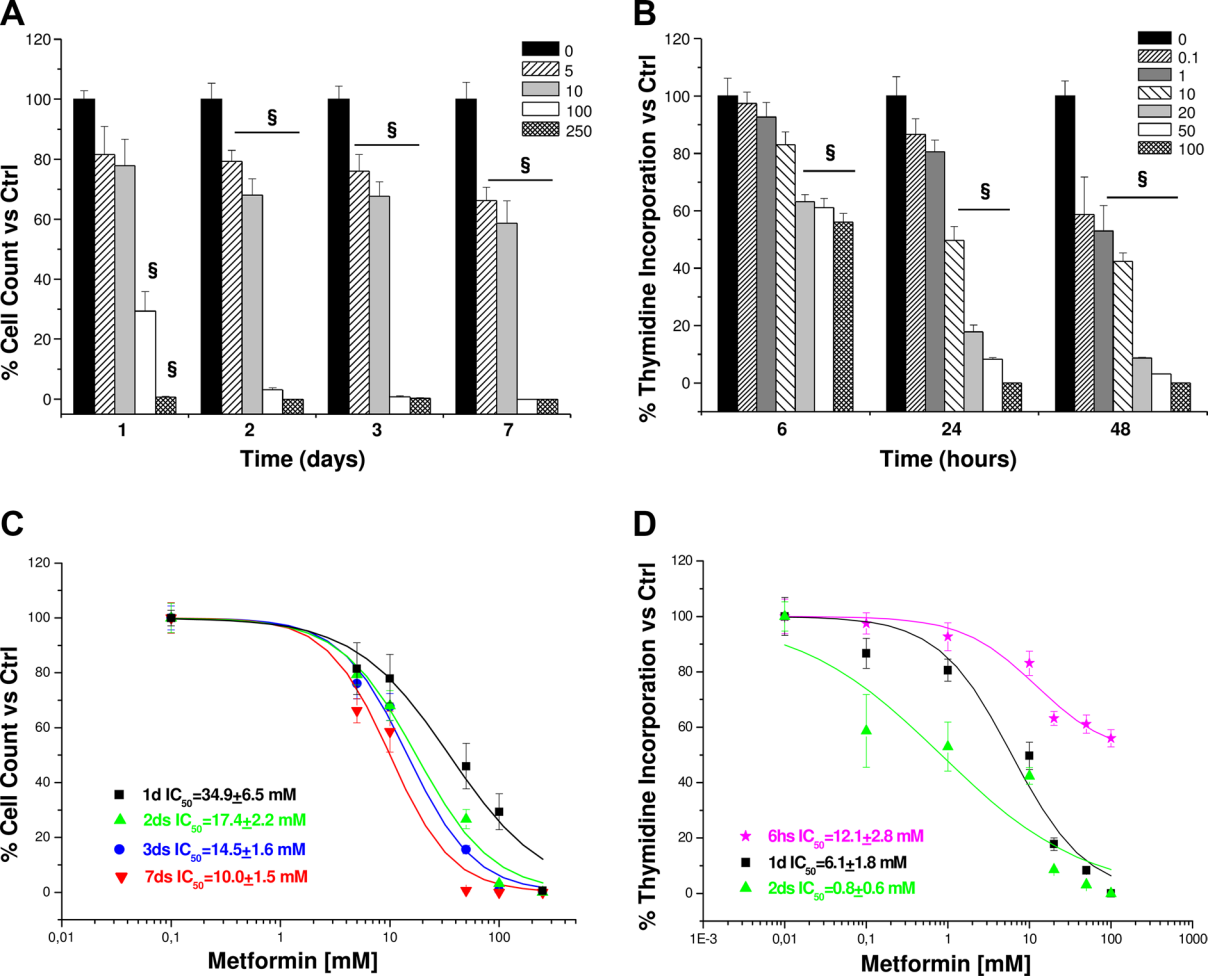
Metformin affects proliferation in H295R cell line Cell proliferation was evaluated by direct cell count and thymidine incorporation (4 hour pulse) in H295R cells treated in absence or presence of increasing concentrations of metformin (mM) at the indicated time points. Data are expressed as mean ± SE of cell count (**A**) or DNA-thymidine incorporation (**B**) percentage vs. non-stimulated controls in *n* =3 independent experiments. Metformin IC50s for cell count (**C**) and thymidine incorporation (**D**) were calculated on the dose-response curves obtained at the indicated time points. Statistical analysis was performed with ANOVA followed by Dunnett's *post hoc* test: ^§^*P* < 0.001 vs. respective controls.

Once shown that metformin significantly affected viability of both cell lines, we chose to focus on the effects in H295R, since this cell model better represents the secreting form of ACC. Inhibitory action of metformin was more pronounced when assessed by direct cell count (Figure 2A) than with MTS analysis; this suggests an additional effect on cell proliferation, as further confirmed by thymidine incorporation assay (Figure 2B). IC_50_s were calculated from dose-response cell count (Figure 2C) and thymidine uptake (Figure 2D) curves for each time point: coherently, calculated IC_50_s decreased with increased treatment time.

Since in non-tumor cells metformin acts as a hypoglycemic drug by facilitating glucose uptake and its utilization, we next evaluated these properties in the H295R cell line and found that metformin dose-dependently stimulated a significant increase in cell basal glucose uptake (Table 1).

**Table 1:** Metformin stimulates glucose uptake in H295R

Metformin (mM)	Glucose uptake (% increase)	SE	*P* value
0	100.0	5.98	-
10	165.6	6.38	0.000
20	161.8	5.21	0.000
50	126.1	3.19	0.002

The 2-deoxy-[^3^H] D-glucose uptake was measured in H295R cells treated for 24 hours with metformin and detected as radioactive counts per minute (CPM) on a scintillation beta counter. Data are expressed as mean ± SE of percentage increase of glucose uptake vs. control without metformin (0 mM). *P* values obtained by statistical analysis performed with ANOVA followed by Dunnett's *post hoc* test vs. control are indicated.

### Metformin inhibits ERK and mTOR signaling in H295R cells

**Figure 3:**
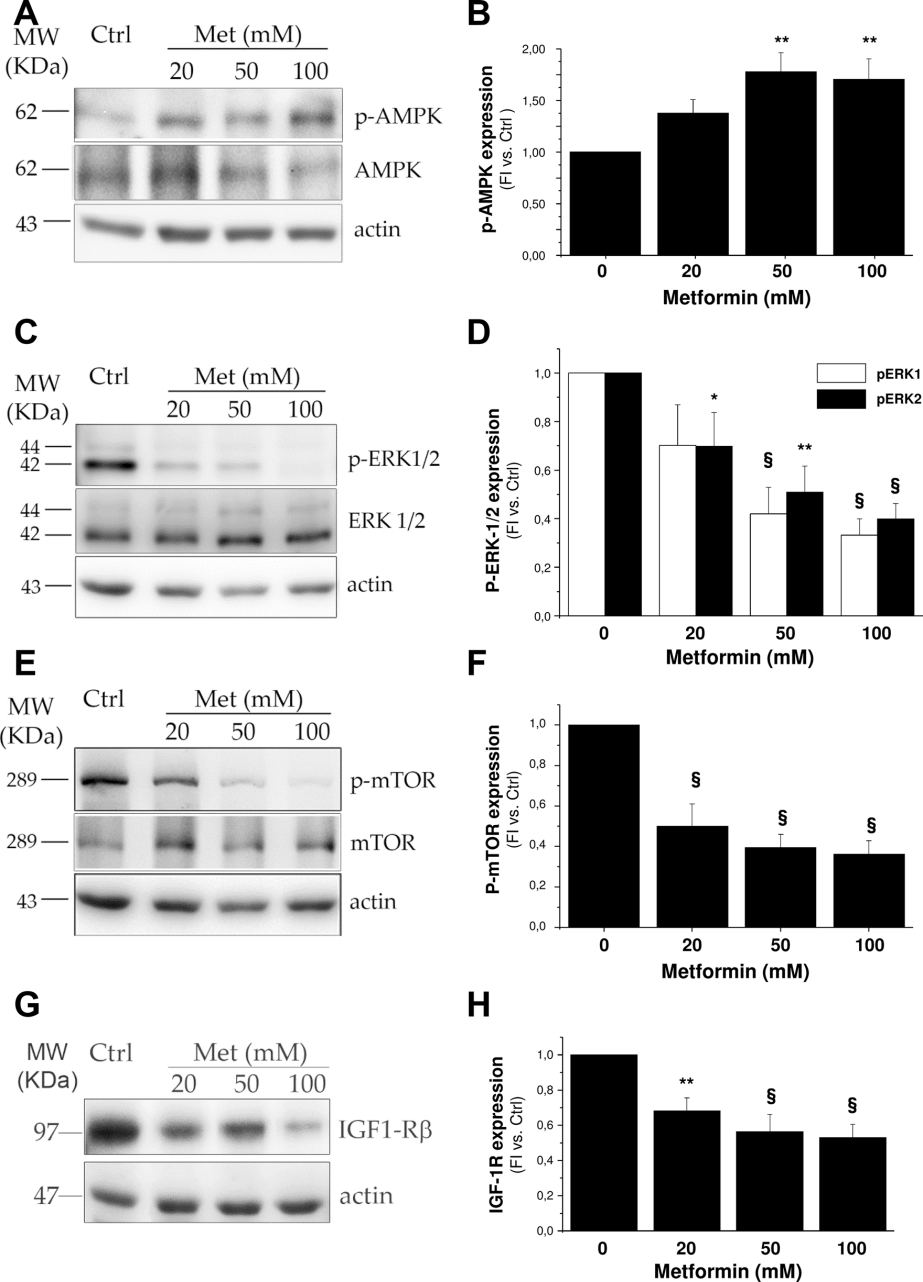
Metformin interferes with ERK and mTOR signaling pathways by activating AMPK Protein extracts from H295R untreated or treated with increasing doses of metformin (20, 50, 100 mM) were analyzed by Western Blot to assess AMPK (**A**) and mTOR (**E**) phosphorylation after 6 hour treatment, while phospho-ERK1/2 (**C**) and IGF-1R (**G**) expression was evaluated after 24 hour treatment. For AMPK, mTOR and ERK 1/2 the total protein forms was also evaluated. Bar charts (**B**, **D**, **F**, **H**) represent mean ± SE band intensity of each protein (p-AMPK, p-ERK1/2, p-mTOR, and IGF-1R, respectively) shown as the respective fluorescence intensity of Western blot bands normalized on actin, which was used as internal loading control, in at least three independent experiments. Statistical analysis was performed with ANOVA followed by Dunnett's *post hoc* test: *P < 0.05,**P < 0.005, ^§^*P* < 0.0001 versus respective controls.

We next investigated the intracellular signaling pathways underlying metformin inhibitory effect on H295R growth. We assessed the ability of the drug to activate the AMP-activated protein kinase (AMPK) energy sensor, via its phosphorylation in the Thr172 residue. Western blot analysis of cell lysates showed a significant dose-related AMPK phosphorylation stimulation, confirming that this intracellular pathway downstream from metformin action is also activated in H295R (Figure 3A, 3B).

Since in colon cancer metformin exerts an anti-proliferative effect by suppressing IGF-1R signaling [10], we next analyzed the activating phosphorylation pattern for Akt and extracellular signal-regulated kinases 1/2 (ERK1/2), the two main IGF-1R downstream pathways in H295R cells [11]. Increasing doses of metformin inhibited phosphorylation of both ERK1 and 2 (Figure 3C, 3D), with no significant effect on Akt phosphorylation (data not shown). Signaling pathways downstream from IGF-1R have been shown to converge in mTOR activation to sustain cell proliferation in both H295R [12, 13] and ACC [14]. A 24 hour metformin treatment induced a dose-dependent inhibition of mTOR activating phosphorylation in the Ser2448 residues (Figure 3E, 3F), as well as a significantly lower IGF-1R net expression (Figure 3G, 3H).

### Metformin activates the apoptotic process in H295R cells

**Figure 4:**
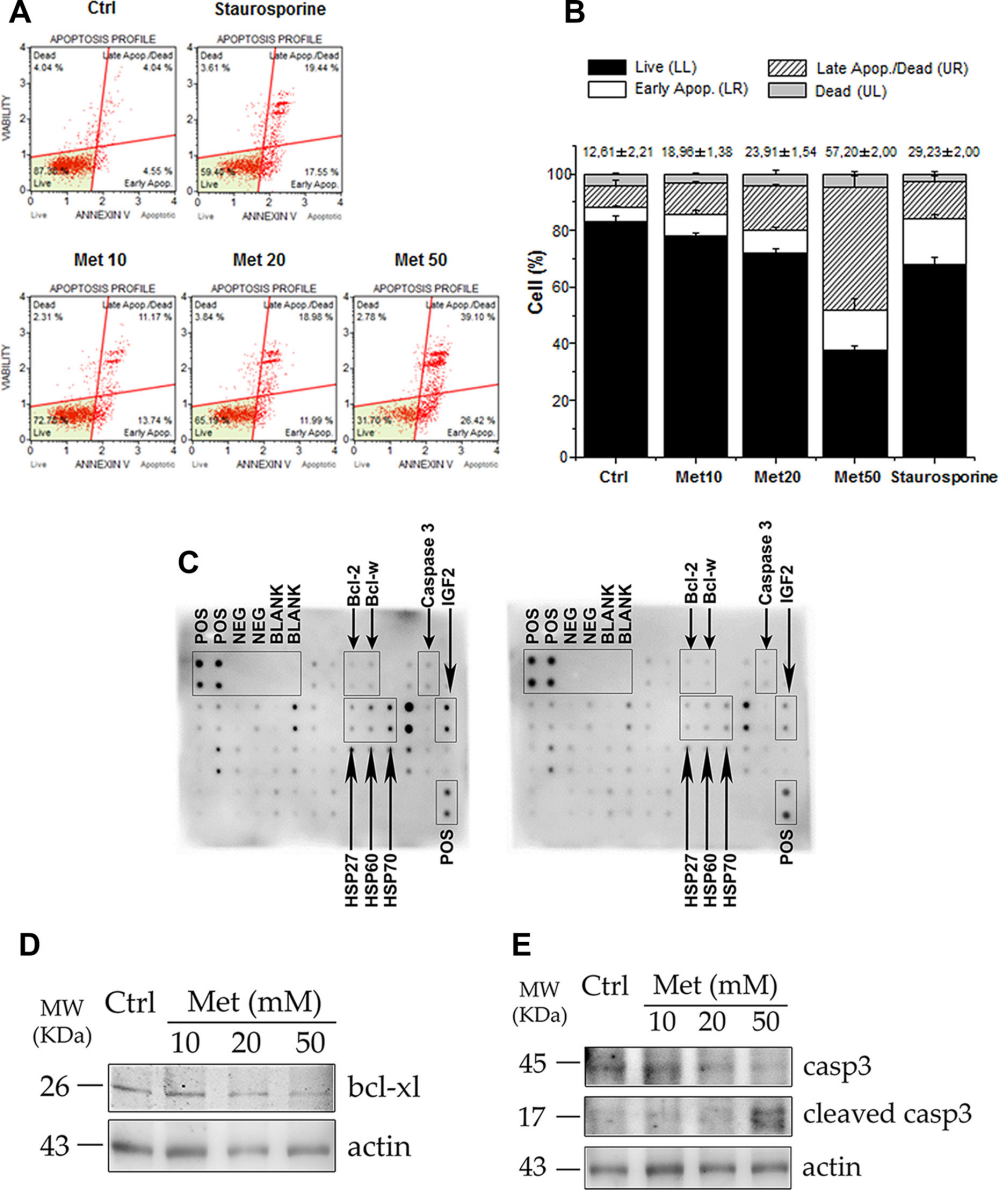
Metformin stimulates apoptosis in H295R cell line (**A**) After 48 hour treatment with increasing doses of metformin (Met 10, 20, 50 mM), H295R cells were trypsinized and analyzed with a Muse automated cell analyzer, using the Muse Annexin V/Dead Cell Assay. This analysis enabled differentiation, on the basis of annexin V positivity, of four populations of cells for each sample: live, early apoptotic, late apoptotic, and dead cells. Cells treated overnight with 0.2 μM staurosporine were used as positive controls of apoptosis induction. (**B**) Bar chart represents mean ± SE of cell percentage for each population identified with Annexin V assay. Mean percentage ± SE of total apoptotic cells related to each sample is also indicated above bar charts. Statistical analysis was performed with ANOVA followed by Dunnett's *post hoc* test: *P < 0.05, **P < 0.001, ^§^*P* < 0.0001 vs respective controls. (**C**) Protein array membranes for apoptosis were incubated with protein extracts from control (left panel) and 20 mM metformin-treated for 48 hours (right panel) cells. Positive and negative spots are indicated, as well as the apoptosis proteins of interest (arrows). (**D**, **E**) Western blot analysis of protein extracts from H295R treated or untreated with the indicated doses of metformin for 48 hours: treated cells show decreased expression of Bcl-xl and an increase in the cleaved active fragments of caspase 3, accompanied by a decrease in the intact form, compared to the control. Actin was used as internal protein loading control.

To investigate whether the reduced number of cells following metformin treatment could be due to an enhanced cell death, we next examined the cascade of events underlying apoptosis in H295R cells. Cytofluorimetric analysis of annexin V exposure (Figure 4A), shows that 48 hour treatment of the cells with increasing doses of metformin (10, 20, 50 mM) stimulates a dose-dependent increase in the percentage of apoptotic cells, both in early and late phases of the apoptotic process, compared with controls (Figure 4A, 4B). This finding is associated with a significant decrease in the number of living cells. Our findings were confirmed by a protein array specifically designed to assess the expression of the main proteins involved in the apoptotic pathway. H295R cells treated with 20 mM metformin for 48 hours expressed lower levels of the anti-apoptotic proteins Bcl-2 and Bcl-w, un-cleaved caspase 3 and heat shock proteins HSP27, HSP60 and HSP70 (Figure 4C). Decreased IGF2 expression was also observed, confirming the inhibitory effect of metformin on the autocrine/paracrine IGF2/IGF-1R system. Western blot analysis of the same protein extracts revealed that 20 mM metformin inhibited Bcl-xl form (Figure 4D) and activated caspase-3 by increasing the cleaved fragments and decreasing the corresponding un-cleaved form (Figure 4E).

### Metformin affects tumor growth *in vivo* in a mouse ACC xenograft model

**Figure 5:**
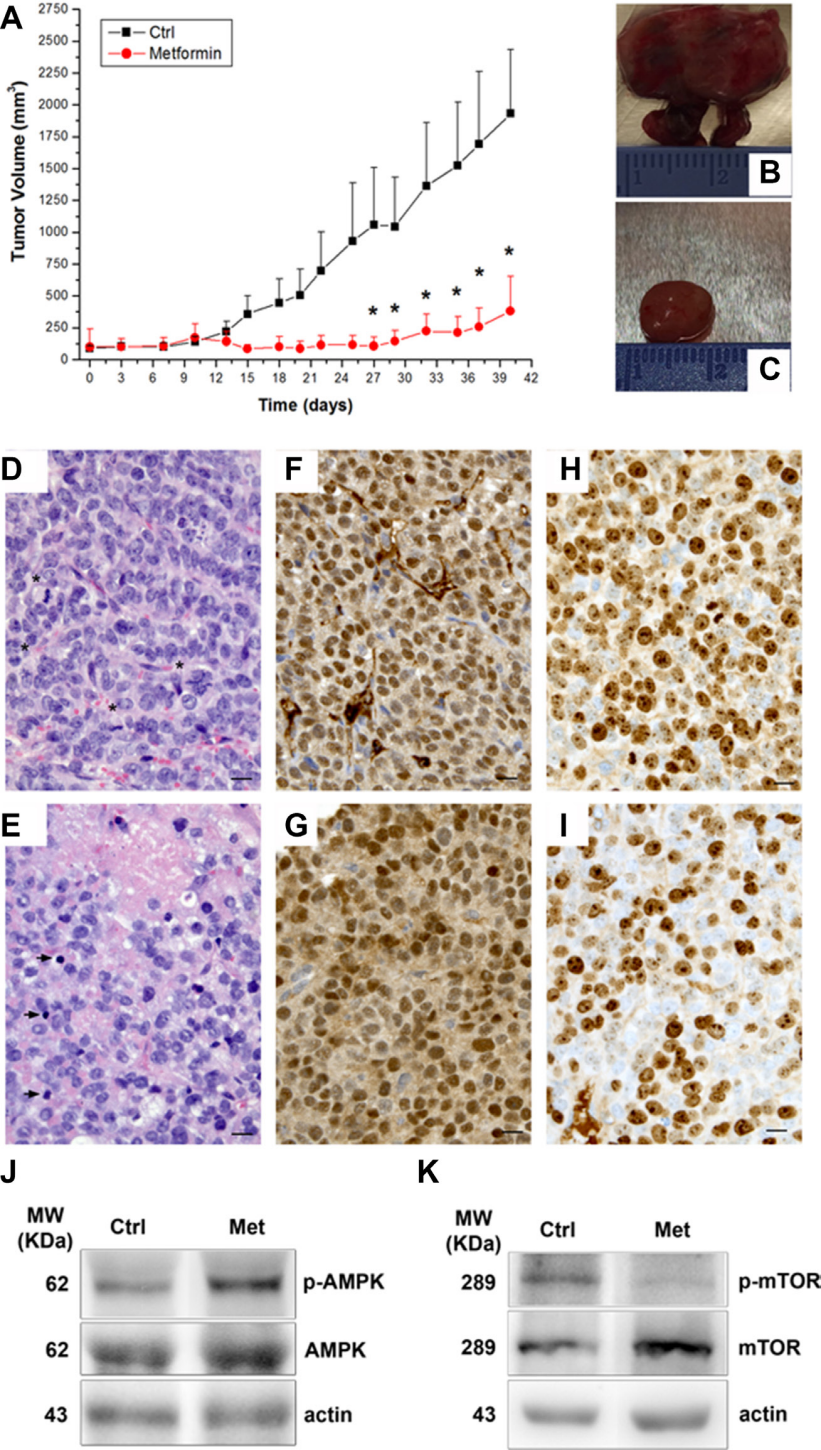
Metformin inhibits tumor growth in a mouse xenograft model of ACC ACC xenografts were obtained by H295R cell subcutaneous injection in CD1 nude mice and, once tumors had reached a detectable 5 mm diameter, animals were randomized to be treated or not with metformin (3 mg/day) for 40 days. Tumor growth was assessed by monitoring the mass volume 3 times a week. (**A**) Tumor growth curves represent mean ± SE of the measured tumor volume over time in control mice (filled squares, *n* = 5) and metformin-treated (filled circles, *n* = 5) groups. *P < 0.05 between the two groups was obtained by Student's *t* test analysis. Analysis of two representative tumors excided after 40 day treatment (end of experiment) from both control and treated mice is given: (**B**, **C**) macroscopic observation; (**D**, **E**: 40× magnification) hematoxylin/eosin staining, asterisks and arrowheads indicate mitotic figures and apoptotic bodies, respectively; (**F**, **G**) immunohistochemistry for SF-1; (**H**, **I**) immunohistochemistry for Ki67; scale bare: 10 μm. Western blot analysis for p-AMPK (J, upper panel) and p-mTOR (K, upper panel) normalized on respective total protein forms (middle panels) and actin (lower panels) of two representative tumors excided from control and metformin-treated mice.

In order to evaluate the *in vivo* metformin effect, we monitored tumor growth in a mouse xenograft ACC model, obtained by subcutaneously injecting H295R cells in the two groups of athymic CD-1 nude mouse strain [15, 16], one treated and one untreated with metformin (3mg/day) for 40 days. Metformin administration was associated with a statistically significant reduced increase in tumor volume after 27 days compared with controls, (Figure 5A). After 15 days of treatment, the inhibitory effect (1-T/C = 76.2 ± 8.4%) approached the maximum (89.8 ± 6.7%), which was reached on day 27th and remained almost constant until the animals were sacrificed (80.2 ± 14.0% inhibition on day 40th).

Macroscopically, excided tumors from the control group were larger, generally lobulated and highly vascularized (Figure 5B) compared with those from the treated group, which were smaller and well-circumscribed (Figure 5C).

Histologically, the tumors from the controls consisted of rather small, uniform cells with coarse chromatin and prominent nucleoli. A haphazard network of small-caliber vessels was also observed (Figure 5D). Mitotic figures, both typical and atypical, were numerous (Figure 5D, asterisks). Conversely, in the metformin-treated group, the tumors showed decreased vascularization, a greater number of apoptotic bodies (arrowheads) and a lower mitotic activity (Figure 5E). Foci of necrosis were occasionally seen (Figure 5E).

Immunohistochemistry demonstrated that tumor cells from both the controls (Figure 5F) and metformin-treated (Figure 5G) mice stained intensely and diffusely with SF-1, confirming that they were H295R-derived. Metformin treatment (Figure 5I) was associated with a reduction in nuclear Ki-67 reactivity, compared to the controls (Figure 5H) (Ki-67 mean ± SEM: 55.1 ± 1.8 vs 74.8 ± 5.2 respectively; *P* < 0.02; 27% inhibition), suggesting an inhibitory effect of metformin on tumor proliferation.

Western blot analysis of protein extracts from excised tumors confirmed the findings observed *in vitro* in H295R (Figure 3A, 3B and 3E, 3F), that metformin-*in vivo* treatment of the xenografted mice was associated with an increased level of p-AMPK (Figure 5J) and a decreased level of p-mTOR (Figure 5K).

## DISCUSSION

Meta-analyses conducted on diabetic subjects treated with metformin suggest a decreased incidence of several types of cancer [6, 7]. Moreover, preclinical studies performed *in vitro* and *in vivo* on different types of solid tumors [8] have shown that metformin also possesses anti-tumor properties. In particular, this drug interferes with the insulin/IGF-1R system in tumor cells [8, 17] as shown in pancreatic [10, 18, 19], breast [20, 21], endometrial [22, 23], prostate [24] and lung [25] cancers. Due to the rarity of ACC, no data are currently available regarding cancer prevalence and metformin treatment in T2D, nor on the effect metformin exerts on tumor growth. Our findings demonstrate that metformin inhibits proliferation in the adrenocortical cancer cell model H295R in association with a decreased expression of the IGF2/IGF-1R system. ACC is an endocrine tumor where the massive secretion of IGF2 acts in an auto/paracrine loop to sustain cancer cell proliferation, via activation of both insulin and IGF-1R pro-survival intracellular pathways [26–28]. In H295R, metformin induces a decrease in IGF2 and IGF-1R expression. The inhibition of IGF-1R promoter activity following metformin treatment could involve activation of p53, pAMPK and E2F1 transcription factor, which among the targets of metformin have been described to regulate IGF-1R gene expression [22, 23, 29].

In H295R cells, we showed that the drug also interferes with the intracellular ERK and mTOR signaling pathways downstream from IGF-1R. We previously demonstrated that another class of anti-diabetic drugs, the thiazolidinediones, including rosiglitazone and pioglitazone, inhibits adrenocortical cancer cell proliferation [11] and stimulates cell differentiation [31, 32]. These drugs also restrained cell proliferation through IGF-1R signaling inhibition. However, while rosiglitazone acts on inhibition of both pathways, metformin seems to mainly affect ERK signaling, with no significant effect on phosphorylation/activation of Akt. A similar mechanism has been described in granulosa cells, where metformin inhibits IGF-1-stimulated cell growth through inhibition of ERK signaling and without affecting Akt [33]. Furthermore, the key role of ERK1/2 in mediating the anti-tumor effect of metformin has been confirmed by a xenograft mouse model using neuroblastoma cell lines, where a reduced ERK1/2 phosphorylation was observed in tumors of metformin-fed mice [34].

The inhibitory effect of metformin on cell growth seems due not only to a reduction in cell proliferation rate, but also to the stimulation of the mitochondrial-dependent apoptotic pathway. Indeed, the complex I of the mitochondrial respiratory chain is one of the main cellular targets of metformin [8]. Metformin inhibition of the oxidative phosphorylation (OXPHOS) process reduces mitochondrial production of ATP, thus inducing cell energy stress and activating the intrinsic apoptotic pathway [8]. In H295R cells, we demonstrated that metformin treatment is associated with a dose-dependent increase of membrane exposure of annexin V, one of the early events in the apoptotic cascade. Protein array analysis also showed a significant decrease in the expression of the anti-apoptotic factors belonging to the family of Bcl-2, the key regulator of the intrinsic apoptotic pathway and of mitochondrial integrity [35]. Induction of apoptosis finally results in the activation of the caspase-3 effector [35], the cleaved fragments of which become detectable in H295R cells following metformin treatment.

Inhibition of OXOPHOS leads to a reduction in the ATP/ADP ratio, thus activating the intracellular key energy sensor AMPK [36, 37]. Activated AMPK mainly interferes with mTOR activity by disrupting its association with mTORC1 [38], finally resulting in a net cytostatic effect. In metformin-treated H295R cells, we observed an increased AMPK phosphorylation, associated with a rapid glucose uptake, which is probably an adaptive compensatory mechanism to fuel ATP production through glycolysis. However, in breast cancer, mTOR inhibition by metformin also blocks glycolytic and tricarboxylic acid cycle intermediates necessary for cancer proliferation [39], thus interfering with the Warburg effect [40]. Similarly, in H295R metformin-induced ERK inhibition and AMPK activation may converge in mTOR blockage. These findings suggest a metabolic switch from a mainly anabolic to a mainly catabolic state to also support energy requirements in the H295R cell line. The kinase protein mTOR acts as a gatekeeper for metabolism and cell growth, catching signals of cell stress, intracellular nutrient levels and growth factors [41]. Besides the PI3K/Akt activation pathway, which seems unaffected by metformin in H295R, mTOR can form an alternative complex with mTORC1 following ERK1/2 activation [42–44], thus specifically mediating the IGF-1/insulin proliferative pathway [12].

Metformin also induces a significant reduction in the expression of heat shock proteins (HSPs) involved in tumorigenesis [45]. The high rate of anabolic processes sustaining cancer cell proliferation and progression in a ROS-rich environment requires a high chaperone activity to ensure a correct protein folding process. Increased HSPs expression has been observed in various types of malignancies [46], including adrenocortical cancer [47]. In particular, HSP27, 60 and 70 play a pivotal role in dampening the apoptotic processes at mitochondrial level [45, 48], thus representing promising anti-tumor targets for the development of anti-cancer drugs. Here, we have demonstrated that metformin treatment leads to reduced HSP27/60/70 expression in H295R cells, which may contribute to the stimulatory effect of the drug on adrenal cancer cell apoptosis.

The *in vitro* anti-proliferative effect of metformin in preclinical models is obtained by using higher doses than those reached in diabetic patients [49], thus apparently limiting its potential use in cancer treatment. However, in some tissues, metformin can accumulate at concentrations several-times higher than those found in the bloodstream [50], as demonstrated for the adrenal gland and liver [51]. The adrenal gland is in fact one of the tissues expressing the highest levels of the organic cation transporters 1 (Oct 1) and 3 (Oct 3) [52], which are responsible for metformin cellular uptake and for its high concentration in this organ [53]. Moreover, due to the high charge of the molecule, metformin specifically concentrates in mitochondria [36]. Thus, it is conceivable that the current metformin dosage used in T2D treatment, despite the micromolar levels reached in the plasma, could reach up to millimolar levels in actively absorbing organs, such as the adrenal cortex. We demonstrated a significant decrease in both tumor growth rate and H295R cell proliferation within the tumor mass in metformin-treated ACC xenografted mice, associated with an increase in AMPK and a decrease in mTOR phosphorylation similar to that observed for *in vitro*-treated H295R. These results were obtained *in vivo* employing a metformin dosage very similar to that used in diabetic patients and in line with the literature on rodent tumor xenograft models [54, 55], supporting the hypothesis of metformin concentration in the ACC tumor.

Several clinical trials, specifically designed with endpoints and outcomes allowing exploration of the anti-cancer properties of metformin, are currently ongoing [https://clinicaltrials.gov]. They will also serve to clarify the doses at which metformin exerts its anti-cancer effects compared with its anti-diabetic properties. Further studies are necessary to evaluate a possible combined therapy with mitotane, also proven to affect mitochondrial function in H295R [56, 57], to reduce the dosage of both drugs together.

In conclusion, our findings provide the first preclinical report on the anti-proliferative and pro-apoptotic effect of metformin in ACC and help to elucidate the intracellular signaling pathways involved. Mitochondrial functions and integrity are also the key targets for the anti-cancer activity of this drug in adrenocortical cancer cells. Further studies are necessary to validate these findings *in vivo* and better clarify the intracellular mechanisms involved in metformin activity, whilst proposing the prospective use of metformin in adrenocortical cancer therapy.

## MATERIALS AND METHODS

### Reagents

Primary antibodies directed against phospho-Thr172-AMPKα1/2 (sc-33524), Actin (sc-1615), IGF-1R*β* (sc-713) were from Santa Cruz Biotechnology, Inc. (Santa Cruz, CA, USA); anti-AMPK-α, anti-phospho-p44/42 ERK1/2 (Thr202/Tyr204), anti-p44/42 ERK1/2, anti-Bcl-xl and anti-mTOR antibodies were from Cell Signaling Technology, Inc. (Danvers, Mass, USA); anti-phospho-Ser2448-mTOR (09-213), and anti-Caspase 3 (AB1899) antibodies were from Merck-Millipore (Darmstadt, Germany). Peroxidase-conjugated secondary antibodies, media and sera for cell cultures and metformin were from Sigma-Aldrich (Milan, Italy). Plastic ware was obtained from Corning (Milan, Italy). MTS solution (CellTiter96^®^ Aqueous One Solution Cell proliferation assay) was from Promega (Madison, WI, USA). [^3^H]-thymidine and 2-deoxy-[^3^H] D-glucose were provided by Perkin Elmer (Waltham, Massachusetts, USA). Other reagents for cell culture and microscopy were obtained from Sigma-Aldrich (Milan, Italy), except where specified.

### Cell cultures

Human ACC cell lines H295R and SW13 were obtained from the American Type Culture Collection (Manassas, VA, USA) and used under passage 20. SW13 were cultured in DMEM/F-12 medium (Sigma-Aldrich) with 10% FBS, 2 mM L-glutamine, 100 U/ml penicillin-100 μg/ml streptomycin, which was further enriched with a mixture of insulin/transferrin/selenium (Sigma-Aldrich) for H295R culturing. Cells were incubated at 37°C in a humidified 5% CO_2_ atmosphere.

### MTS assay

H295R and SW13 cells seeded in 96-well plates (1 × 10^4^ and 2.5 × 10^3^ cells/well, respectively) were 24-hour starved and treated in 10% FBS-medium with vehicle (control) or increasing metformin doses for the indicated time points. Media were replaced every three days. Cell viability was assessed by MTS assay, according to the manufacturer's instructions, and analyzed by an ELISA plate reader (Wallac 1420, PerkinElmer, Monza, Italy) at 490 nm wavelength to measure optical density (OD). Each experimental point was performed in six replicates in at least three independent experiments.

### Viable cell count

H295R cells seeded in 12-well plates (1 × 10^5^ cells/well) were 24 h-starved and treated in 10% FBS-medium with vehicle (control) or increasing doses of metformin for the indicated time points. At each time point, cells were trypsinized and counted by a haemocytometer, after dead cell exclusion with trypan blue staining. The mean cell number was obtained by counting four replicates in three different experiments.

### DNA synthesis assay: [^3^H]-thymidine (TdR) incorporation

DNA synthesis was evaluated according to the amount of [^3^H]TdR incorporated into trichloroacetic acid (TCA)-precipitated materials. Cells grown in 10% FBS-complete medium till 70% confluence, were starved for 24 hours and treated with increasing doses of metformin for 6, 24, 48 hours, pulsing them with 0.5 μCi/ml [^3^H] TdR (6.7 Ci/mmol) for 4 hours before halting proliferation in ice-cold 10% TCA. After washing in 5% TCA, cells were solubilized in 0.25 *N* NaOH and radioactivity was measured using a scintillation beta counter. Each experimental point was performed in four replicates in at least three independent experiments.

### Glucose uptake measurement

H295R cells seeded in 12-well plates (1 × 10^5^ cells/well) and grown up to confluence, were washed twice with PBS and t incubated overnight in a serum free, low glucose medium (0.55 mM). Cells were treated in the absence (control) or presence of increasing doses of metformin for 24 hours. After PBS-wash, cells were incubated with Hepes buffer (140 mM NaCl, 20 mM Hepes-Na pH 7.4, 2.5 mM MgSO4, 1 mM CaCl2, 5 mM KCl) containing 2-deoxy-[^3^H]D-glucose [1μCi/μl] for 10 minutes at 37°C. After wash with cold PBS, cells were lysed in 100 mM NaOH for 1 hour at 37°C. Radioactivity was measured by a scintillation beta counter.

### SDS-PAGE and western blot analysis

Treated cells were lysed in RIPA buffer (20 mM Tris, pH 7.4, 150 mM NaCl, 0.5% Triton-100, 1 mM Na3VO4, 1 mM PMSF) and, after protein measurement by Comassie method, equal amounts of proteins for each sample (30 μg) were separated by SDS–PAGE and transferred onto PVDF membranes (Immobilon, Merck Millipore). Each membrane was incubated overnight at 4°C with primary antibodies at the appropriate dilutions, then with peroxidase-secondary IgG (1:2000) at room temperature for 1 hour. Image acquisition and densitometric analysis were performed with Quantity One software on a ChemiDoc XRS instrument (BIO-RAD Labs, CA, USA). All Western blots were repeated in at least 3 independent experiments. Actin was used as internal loading control to normalize protein expression.

### Apoptotic evaluation by Muse™ cytofluorimetric analysis

After 72 h treatment with increasing doses of metformin (0, 10, 20, 50 mM), H295R cells were trypsinized and analyzed with a Muse^™^ automated cell analyzer (Merck Millipore, Billerica, MA, USA) for apoptosis detection using the Muse™ Annexin V/Dead Cell (cat # MCH100105, Merck Millipore, Billerica, MA, USA) assay, according to the manufacturer's instructions. Cells treated for 2 hours with 2 μM staurosporine were used as positive control for apoptosis induction.

### Apoptosis antibody array

Cell lysates from H295R cells treated or untreated with 20 mM metformin for 48 hours were analyzed using a human apoptosis antibody array (RayBiotech, Norcross, GA, USA) according to the manufacturer's instructions. Array spot emission images were captured by ChemiDoc XRS instrument (BIO-RAD Labs, CA, USA).

### Xenograft model for tumor growth assessment

Female athymic CD1 nude mice (9 week-old, Charles River Laboratories, Italy) were inoculated subcutaneously with H295R cell suspension (7 × 10^6^ cells/100 μl). Almost all H295R injected mice developed a detectable tumor except one (84% overall tumor take rate), confirming the aggressiveness of ACC derived cells. Tumor growth was monitored daily and once solid tumors reached a 5 mm mean diameter the animals were randomly assigned to intraperitoneal injection of metformin (3 mg in 100 μl PBS/day, 6 days/week) or vehicle. Tumor volume (mm3) was monitored 3 times a week by two independent investigators and was calculated by using the following formula: length x width^2^/2. Anti-tumor activity of the drug was evaluated in terms of tumor growth inhibition percentage, calculated by 1-Treated/Control tumor volume ratios (1-T/C) [58]. Data were expressed as mean ± SE.

Drug tolerability in tumor-bearing mice was assessed in terms of: a) lethal toxicity, i.e. any death in treated mice occurring before any death in control mice; b) body weight loss percentage = 100 − (body weight on day x/body weight on day 1) × 100, where x represents a day after or during the treatment period [58].

Animal studies were performed in compliance with an institutionally approved protocol and with the National Institutes of Health Guide for the Care and Use of Laboratory Animals.

### Histological and immunohistochemical examination of xenografts

Tissues were fixed in 10% buffered formalin and paraffin-embedded. Five-micrometer sections were hematoxylin and eosin (H/E) stained for histologic evaluation or used for immunohistochemistry. Immunohistochemical analysis with mouse anti-human Ki-67 monoclonal (Dako, Glostrup, Denmark) was performed with the Ventana Benchmark XT system (Ventana Medical Systems, Tucson, AZ, USA). Nuclei were hematoxylin counterstained. Ki-67 positive nuclei were counted on 1000 tumor cells in 3 tumors for each group. Negative controls were performed by omitting the primary antibodies. Immunohistochemical analysis with rabbit anti-human SF-1 polyclonal IgG (Upstate, Charlottesville, VA) was performed with the Ventana Benchmark XT system (Ventana Medical Systems, Tucson, AZ, USA).

### Statistical analysis

Statistical analysis was performed using SPSS 22.0 software (SPSS Inc. Chicago, IL, USA). The Kolmogorov–Smirnov test was used to verify the normal distribution of data which were then expressed as mean ± SE. One way ANOVA followed by post-hoc Dunnett's test was applied for multiple comparisons, while Student's *t* test was employed for comparing the two classes of data shown in figures. A *P* < 0.05 value was considered statistically significant. The half Inhibitory Concentration (IC_50_) of metformin was calculated on cell count, MTS and thymidine-incorporation dose-response curves using Origin software 6.1 version (OriginLab Corporation, Northampton, MA).
